# Development of a Head-Mounted Holographic Needle Guidance System for Enhanced Ultrasound-Guided Neuraxial Anesthesia: System Development and Observational Evaluation

**DOI:** 10.2196/36931

**Published:** 2022-06-23

**Authors:** Jaya Tanwani, Fahad Alam, Clyde Matava, Stephen Choi, Paul McHardy, Oskar Singer, Geraldine Cheong, Julian Wiegelmann

**Affiliations:** 1 Department of Anesthesiology and Pain Medicine Sunnybrook Health Sciences Centre University of Toronto Toronto, ON Canada; 2 Department of Anesthesia The Hospital for Sick Children University of Toronto Toronto, ON Canada; 3 Department of Anesthesia Khoo Teck Puat Hospital Singapore Singapore

**Keywords:** mixed reality, virtual reality, augmented reality, HoloLens, holograms, neuraxial anesthesia

## Abstract

**Background:**

Neuraxial anesthesia is conventionally performed using a landmark-based technique. Preprocedural ultrasound is often used in challenging clinical scenarios to identify an ideal needle path. The procedure is then carried out by the operator recreating the ultrasound needle path from memory. We suggest that a needle guidance system using the Microsoft HoloLens mixed reality headset, which projects a hologram of the ideal needle path, can assist operators in replicating the correct needle angulation and result in fewer needle passes.

**Objective:**

The objective of the study was to develop software for the mixed reality HoloLens headset, which could be used to augment the performance of neuraxial anesthesia, and establish its face validity in lumbar spine phantom models.

**Methods:**

We developed an ultrasound transducer marker and software for the HoloLens, which registers the position and angulation of the ultrasound transducer during preprocedural scans. Once an image of a clear path from skin to the intrathecal space is acquired, a hologram of the ideal needle path is projected onto the user’s visual field. The ultrasound probe is removed while the hologram remains in the correct spatial position to visualize the needle trajectory during the procedure as if conducting real-time ultrasound. User testing was performed using a lumbar spine phantom.

**Results:**

Preliminary work demonstrates that novice (2 anesthesia residents) and experienced operators (5 attending anesthesiologists) can rapidly learn to use mixed reality holograms to perform neuraxial anesthesia on lumbar spine phantoms.

**Conclusions:**

Our study shows promising results for performing neuraxial anesthesia in phantoms using the HoloLens. Although this may have wide-ranging implications for image-guided therapies, further study is required to quantify the accuracy and safety benefit of using holographic guidance.

**Trial Registration:**

ClinicalTrials.gov NCT04028284; https://clinicaltrials.gov/ct2/show/NCT04028284

## Introduction

Neuraxial anesthesia has traditionally been a landmark-based technique, relying on operator feel, skill, and experience. Difficulty is highly influenced by patient body habitus, where obese patients or anatomical variations such as scoliosis or osteophytes increase difficulty and result in a higher failure rate [1,2]. Neuraxial anesthesia is not a benign procedure as multiple attempts or inaccurate needle trajectories can be anxiety provoking, cause patient discomfort, and lead to morbidity in the form of spinal/epidural hematomas, infection, dural puncture with a high risk of subsequent headaches, and nerve injury [3].

Though increasing in popularity, ultrasound guidance for neuraxial procedures is still relatively uncommon secondary to technical challenges of real-time guidance in conjunction with the difficulties of ultrasound imaging of bony structures [4]. In contrast to ultrasound-guided peripheral nerve blocks done with real-time guidance where the needle tip is visualized, the common technique for ultrasound use in neuraxial anesthesia is to provide preprocedure landmarks so the operator estimates the placement of the needle tip, depth, and trajectory before needle insertion. Anatomical landmarks are visualized using the ultrasound along multiple viewing planes and skin markings are made based on these images. The ultrasound probe is then removed from the site, placed at rest and subsequent needle insertion is done blindly based on the skin markings and the provider’s recollection of approximate depth and trajectory from memory. This is not true ultrasound guidance per se, but rather ultrasound-assisted guidance. Little is known about the accuracy with which operators replicate an ideal needle path once identified via ultrasound.

The Microsoft HoloLens was introduced in 2016 and is the first self-contained, head-mounted mixed reality (MR) computing device. The headset is equipped with 4 tracking cameras and an infrared time-of-flight sensor, which allow 3D mapping of the surrounding environment, objects, and the user’s hands in real time. It allows for MR, positionally stable holograms projected into a user’s visual field. The user interface allows the detection of intuitive hand gestures or voice commands for application control [5]. Medical applications have included education, remote consultation, preoperative surgical planning, and surgical/procedural navigation [6]. We aimed to develop a proof-of-concept MR solution using the HoloLens to aid neuraxial blockade by allowing visualization of the ideal needle path identified on preprocedure ultrasound.

## Methods

### Ethical Considerations

This pilot study is part of a randomized controlled trial titled “Using Augmented Reality to 3D Map Needle Pathways in Real Time to Enhance Neuraxial Anesthesia,” which has been approved by the Sunnybrook Research Institute Ethics Board (#291-2018). Study objectives and protocol were explained in detail to eligible participants (anesthesia residents and attendings), after which both verbal and written consent were obtained.

### Hardware Configuration and Software Development

**Figure 1:**
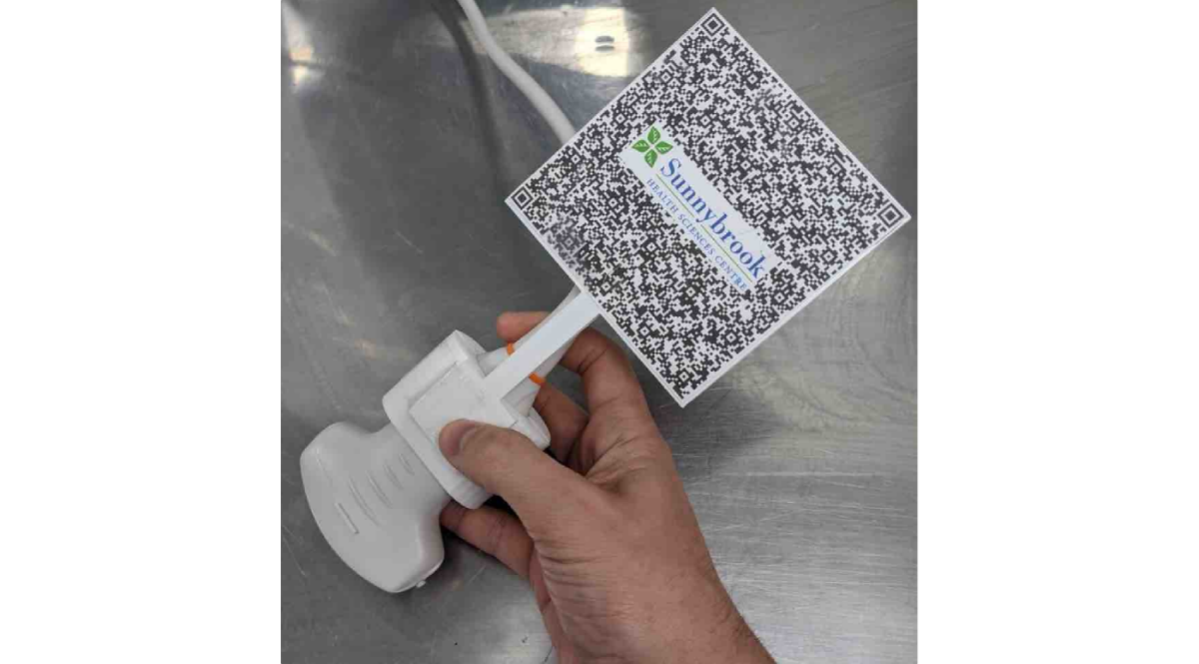
Optical tracking marker attached to a curvilinear probe to enable the HoloLens to detect transducer position.

To enable the HoloLens to detect the ultrasound transducer position, a quick response code optical tracking marker was developed (Figure 1). First, a high-resolution 3D scan of a curvilinear ultrasound probe was carried out with an EinScan HX handheld 3D scanner (SHINING 3D Tech Co Ltd). The resulting model was modified via Autodesk Fusion 360 (Autodesk Inc., San Rafael, United States of America), a computer aided design software to create a probe mount which matched the shape of the US transducer and allowed the attachment of a 10 cm by 10 cm two-dimensional barcode. We found this to be the smallest size barcode that was reliably registered by the HoloLens’s 2-megapixel camera [5]. The marker was then 3D-printed via a CR-10S 3D printer (Creality) using polylactic acid material.

**Figure 2:**
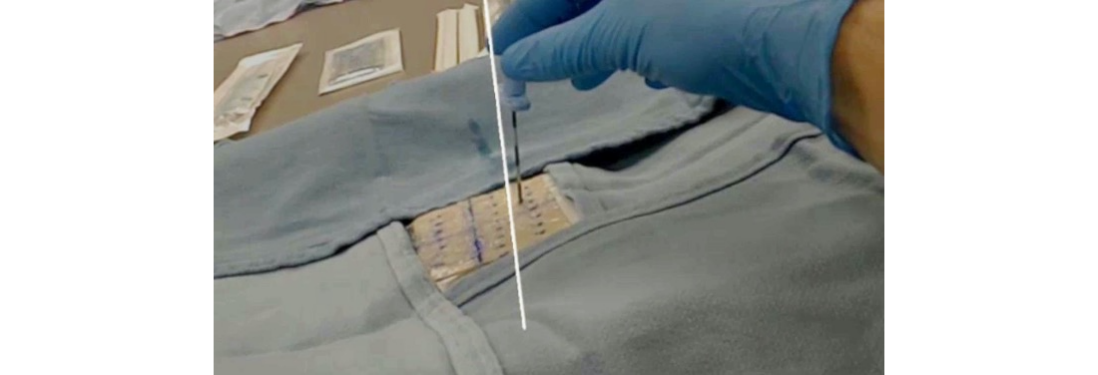
This figure depicts a HoloLens user's view of aligning a Tuohy needle with a hologram representing an ideal needle path (white line) into an ultrasound phantom. The difference between the Tuohy needle and hologram positions is due to a recording artifact and is not perceived by the user.

Software development was conducted in the Unity development environment (Unity Technologies). The software will be made open source following completion of future studies. Detection of the 2D barcode was accomplished by incorporating the Vuforia Augmented Reality (AR) Software Development Kit (PTC Inc). The software allows the HoloLens headset to precisely register the position of the barcode, and therefore the ultrasound transducer, via the tracking marker. Upon registering the ultrasound marker location, the operator then confirms on the ultrasound screen that the desired trajectory is displayed. The trajectory hologram is frozen by putting the ultrasound probe and marker outside the HoloLens camera visual field. We have found that the ultrasound probe marker’s position is registered by the HoloLens in most configurations that are ergonomic for ultrasound use. A hologram of a line is then projected into the headset user’s visual field in the location of the central axis of the ultrasound transducer (Figure 2). The position, angulation, and size of the needle path hologram remain constant as the HoloLens operator moves. Likewise, if the patient moves, the needle path hologram does not change position and hence the patient must return to their original position to maintain the accuracy of the previously identified ideal needle path. Of note, the hologram does not provide any visual projection of the optimal depth.

To establish feasibility, a lumbar spine neuraxial phantom was created for pilot testing of the guidance system [7]. Five attending anesthesiologists and two anesthesia residents were recruited. They were given a 5-minute orientation to the developed MR needle guidance system. After the orientation, we allowed an unlimited amount of time to practice using the HoloLens technology on lumbar spine neuraxial phantoms.

Multimedia Appendix 1 provides an in-depth look at how the HoloLens is used for guidance of needle angulation in phantom models. The difference between the Tuohy needle and hologram positions is due to a recording artifact and is not perceived by the user.

### Augmented Procedural Technique Using MR

**Figure 3:**
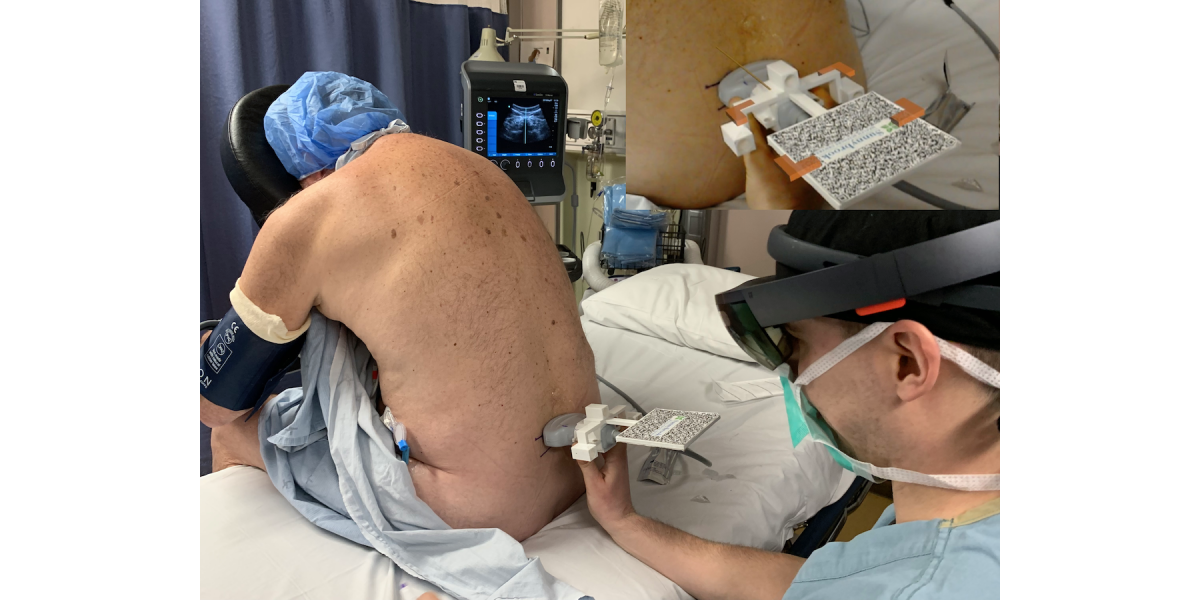
The user performs a preprocedural ultrasound, allowing the headset to subsequently generate a spatially stable hologram. The inset shows the operator's mixed reality view displaying a holographic guidance graphic (orange line along the central axis of the ultrasound transducer) with the tracking marker attached to the ultrasound probe.

Similar to established techniques, the patient is optimally positioned for neuraxial anesthesia. The sitting or lateral decubitus position may be used. A patient positioning device is ideal to minimize patient movement. The operator performs the procedure while wearing the HoloLens headset, which minimally interferes with procedure ergonomics and visibility. A preprocedural, nonsterile ultrasound scan of the lumbar spine is performed with the prepared tracking marker attached to the transducer (Figure 3). The posterior complex is identified and placed in the middle of the ultrasound screen. The angulation of the ultrasound transducer is then registered by the headset detecting the position of the ultrasound transducer marker, and a hologram is projected into the user’s workspace, which replicates the needle path through the middle of the transducer (Figure 3 inset) in a clear path from skin to posterior complex. The central axis of the ultrasound transducer is represented by a holographic white or orange line (10 mm length, 2 mm diameter). These steps can be adapted for a paramedian approach.

**Figure 4:**
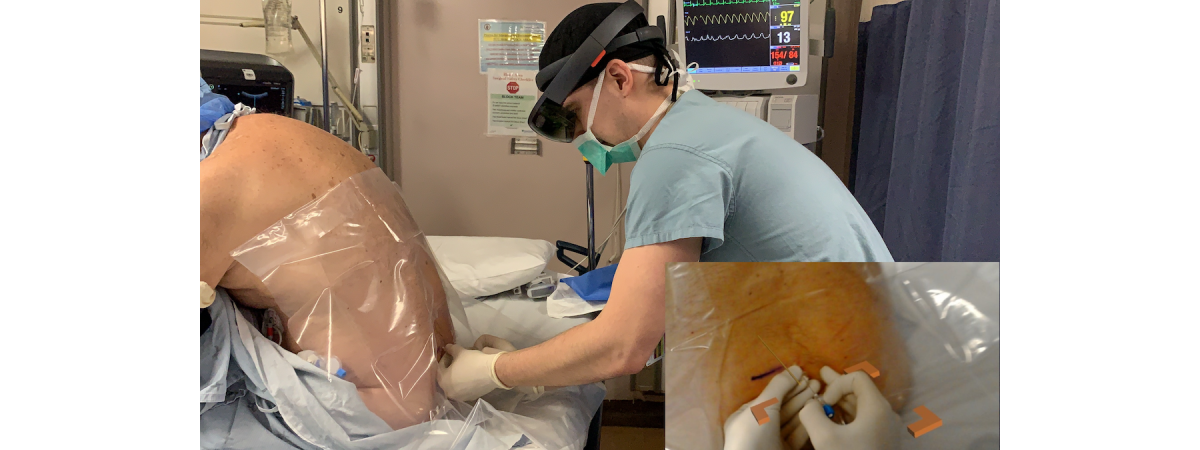
The user performs a neuraxial technique in standard sterile fashion. The inset shows the hologram projected into the user's visual field to aid replication of the ideal needle path as identified on ultrasound.

The transducer is removed from the field, and its center point is marked on the skin in the usual fashion. The spatially stable hologram representing the ideal needle path remains projected into the user’s visual field although the hologram does not provide any information on the desired needle depth nor does it register the needle’s position in the user’s visual frame. Typical sterile prepping and draping and local anesthetic injection do not disrupt the position of the needle path hologram. Operators may then use this hologram to precisely align the needle angulation with the holographic projection in 3 dimensions from the marked skin entry point (Figure 4).

## Results

After an informed consent process, 7 participants were recruited for the study. Five were attending anesthesiologists with a minimum of 2 years of clinical experience as consultants and two were anesthesia residents (postgraduate year 3 or above). Upon completion of the orientation and practice session, participants were asked a yes or no question: “Do you feel adequately prepared and comfortable to use this needle guidance system with a patient?” All participants indicated “yes.” All participants required 3 or fewer practice repetitions with the lumbar spine neuraxial phantoms to feel comfortable.

## Discussion

### Principal Findings

To our knowledge, this study is the first description of the development and feasibility testing of an MR tool for neuraxial anesthesia using a head-mounted display. We developed holographic needle guidance software using the Microsoft HoloLens and determined its use to be feasible in lumbar spine phantoms. Since not all procedures are amenable to real-time ultrasound due to challenges of simultaneously scanning and performing the technique (such as in the case of neuraxial blockade), establishment of the optimal midline and trajectory can be crucial for procedure success. Our MR system allows for a trajectory aid in such situations.

Multiple studies support the use of MR and/or AR systems for various procedures. Ameri et al [8] designed an MR ultrasound image-guided system specifically for internal jugular vein central line insertion. Their system provided a virtual depiction of the needle and its trajectory throughout the procedure. They found that using this system led to a higher success rate of central line insertions in phantom models compared to ultrasound guidance alone for novice users (graduate students). However, when the system was used by 25 experienced physicians (attendings, residents, and fellows from anesthesiology, critical care, and emergency medicine), there was no benefit compared to ultrasound alone.

In an interventional radiology setting, Faiella et al [9] used a similar AR navigation system for computed tomography–guided percutaneous lung biopsies. The system used separate sensors to track needle position and orientation as well as patient movement. Their group found the system easy to use and diagnostically accurate with a low complication rate. This system was particularly efficacious for pulmonary nodules less than 10 mm in size, for which they noted the most drastic reduction in procedure time and a greater proportion of histological diagnoses obtained. Also in an interventional radiology setting, Marker et al [10] successfully used an AR-navigated interventional magnetic resonance imaging system for perineural injections of the thoracic, lumbar, and hypogastric sympathetic plexi, quoting a mean needle tip error of 3.9 (SD 1.7) mm and a mean procedure time of 33 (SD 12) minutes. Similar to our study, their system provided an ideal needle trajectory but did not track the needle in real time. Cumulatively, these studies demonstrate the efficacy of MR/AR systems for multiple procedure types and suggest potential for increased procedural efficacy and efficiency.

However, various limitations exist for the use of MR/AR for medical procedures. In a study of 17 participants comparing the use of AR with ultrasound alone, there was no statistically significant difference in the accuracy of identifying spinal levels prior to epidural placement between the two modalities, suggesting that AR may not enhance procedure accuracy [11]. A 2018 study by Condino et al [12] compared 20 participants’ performance on connect-the-dots tasks using the Microsoft HoloLens versus the naked eye on monocular and binocular trials. Although participants rated task workload and visual comfort as being similar between modalities, user performance was statistically superior during naked eye trials. This group concluded that AR devices may not increase the precision of manual tasks.

Other challenges presented by the use of MR/AR in clinical settings, especially for new users, include visual field distortion, attentional blindness, and challenges with software user interface manipulation (especially while conducting a sterile procedure) [13]. Cost is also a potential barrier to accessing these technologies. The most recent version of the Microsoft HoloLens is currently retailing for US $3500 [5].

Although this study shows promising results, further data are needed to investigate the effectiveness of MR use for neuraxial blockade. Our group is currently conducting a randomized controlled trial to compare traditional techniques for thoracic epidural placement to an MR/HoloLens-assisted technique for elective abdominal surgery at Sunnybrook Health Sciences Centre (ClinicalTrials.gov identifier NCT04028284).

### Conclusions

In this study, we report the successful development and first use of an MR needle guidance technique for neuraxial anesthesia using a head-mounted device, the Microsoft HoloLens. Although this may have wide-ranging implications for many image-guided therapies, further study is required to quantify the potential accuracy and safety benefit of holographic guidance.

## Appendix

Multimedia Appendix 1 Holographic guidance of needle angulation in a phantom model of the lumbar spine.

## Abbreviations

AR: augmented reality
MR: mixed reality

## References

[1] de Oliveira Filho GR, Gomes HP, da Fonseca MHZ, Hoffman JC, Pederneiras SG, Garcia JHS (2002). Predictors of successful neuraxial block: a prospective study. European Journal of Anaesthesiology.

[2] Sprung J, Bourke D, Grass J, Hammel J, Mascha E, Thomas P, Tubin I, McGrady E (1999). Predicting the Difficult Neuraxial Block. Obstetric Anesthesia Digest.

[3] Horlocker TT (2000). Complications of Spinal and Epidural Anesthesia. Anesthesiology Clinics of North America.

[4] Perlas A, Chaparro L, Chin K (2016). Lumbar Neuraxial Ultrasound for Spinal and Epidural Anesthesia: A Systematic Review and Meta-Analysis. Reg Anesth Pain Med.

[5] Microsoft.

[6] Hu H, Feng X, Shao Z, Xie M, Xu S, Wu X, Ye Z (2019). Application and Prospect of Mixed Reality Technology in Medical Field. Curr Med Sci.

[7] Mashari A, Montealegre-Gallegos M, Jeganathan J, Yeh L, Qua Hiansen J, Meineri M, Mahmood F, Matyal R (2018). Low-cost three-dimensional printed phantom for neuraxial anesthesia training: Development and comparison to a commercial model. PLoS One.

[8] Ameri G, Baxter J, Bainbridge D, Peters T, Chen E (2018). Mixed reality ultrasound guidance system: a case study in system development and a cautionary tale. Int J Comput Assist Radiol Surg.

[9] Faiella E, Frauenfelder G, Santucci D, Luppi G, Schena E, Beomonte Zobel B, Grasso R (2018). Percutaneous low-dose CT-guided lung biopsy with an augmented reality navigation system: validation of the technique on 496 suspected lesions. Clin Imaging.

[10] Marker D, U Thainual P, Ungi T, Flammang AJ, Fichtinger G, Iordachita II, Carrino JA, Fritz J (2017). 1.5 T augmented reality navigated interventional MRI: paravertebral sympathetic plexus injections. Diagn Interv Radiol.

[11] Al-Deen Ashab H, Lessoway V, Khallaghi S, Cheng A, Rohling R, Abolmaesumi P (2013). An Augmented Reality System for Epidural Anesthesia (AREA): Prepuncture Identification of Vertebrae. IEEE Trans Biomed Eng.

[12] Condino S, Carbone M, Piazza R, Ferrari M, Ferrari V (2020). Perceptual Limits of Optical See-Through Visors for Augmented Reality Guidance of Manual Tasks. IEEE Trans Biomed Eng.

[13] Kobayashi L, Zhang X, Collins S, Karim N, Merck D (2018). Exploratory Application of Augmented Reality/Mixed Reality Devices for Acute Care Procedure Training. West J Emerg Med.

